# Quantifying mortality burden in patients with cancer due to COVID‐19 in the US: A national cross‐sectional analysis

**DOI:** 10.1002/cam4.6364

**Published:** 2023-08-03

**Authors:** Bhaghyasree Jambunathan, Jacob Lang, Malik Mays, Obi Ekwenna

**Affiliations:** ^1^ The University of Toledo College of Medicine and Life Sciences Toledo Ohio USA; ^2^ Department of Urology and Transplantation The University of Toledo College of Medicine and Life Sciences Toledo Ohio USA

**Keywords:** cancer risk factors, epidemiology and prevention, medical oncology

## Abstract

**Introduction:**

There is limited information on the impact of certain social factors on mortality outcomes in patients with cancer and COVID‐19 on a national scale. This study aims to characterize excess mortality and analyze a subset of sociodemographic trends in COVID‐19 and cancer mortality.

**Methods:**

Patients with cancer listed on their death certificates from 2018 to 2021 and patients with COVID‐19 and cancer listed on multiple cause of death certificates from the CDC Wide‐Ranging Online Data for Epidemiologic Research database from March 2020 to December 2021 were included. Age‐adjusted mortality rates (AAMR) per 1,000,000 population were compared across race/ethnicity groups, sex, and census regions. Crude mortality rates were compared across different age groups and regions based on urbanization status.

**Results:**

Average AAMR in patients with COVID‐19 and cancer was 41.7 in 2020 and 56.7 in 2021.

**Conclusions:**

Mortality rates in patients with cancer and COVID‐19 were significantly higher in certain populations. Targeted interventions are necessary to improve outcomes.

## 1 INTRODUCTION

Persons with cancer have significantly higher risk of adverse outcomes and mortality due to COVID‐19.^1^ Inherent in this relationship are associations with underlying risk factors including higher burden of comorbidities, older age, immunosuppression due to chemoimmunotherapy use, and need for more frequent clinical visits for follow‐up appointments and treatments.^1^, ^2^, ^3^ In addition, patients with cancer during the pandemic experienced more problems with access to care due to a variety of reasons, such as difficulty in obtaining timely appointments due to the healthcare shifts in focusing on COVID‐19 specific care, leading to potential delayed diagnosis and treatments.^4^ Sociodemographic factors such as zip code of residency, non‐Hispanic (NH) Black race/ethnicity, and male sex have also been associated with hospitalization and mortality, however, their study has been limited in the pandemic.^5^ Han et al. quantified deaths due to COVID‐19 and cancer using data from 2020, prior to the widespread availability of vaccination, and found that factors such as American Indian or Alaskan Native, Black, and Hispanic race/ethnicity, age greater than or equal to 85, and residence in a large central metropolitan area were significant risk factors for mortality in patients with cancer and COVID‐19.^6^ This study aims to expand upon this data by (1) characterizing excess mortality due to COVID‐19 compared to prepandemic levels using data from 2018 to 2021 and (2) analyzing sociodemographic trends, particularly in race/ethnicity groups, sex, census regions, urbanization status, and age groups, in mortality rates in patients with COVID‐19 and cancer with newly available 2021 data to assess the effect of widespread vaccine availability on this relationship.

## 2 METHODS

The Multiple Cause of Death (MCOD) CDC Wide‐Ranging Online Data for Epidemiologic Research (WONDER) database was queried for de‐identified data on patients with malignant neoplasms (ICD‐10 C00‐C97) listed on their death certificates from 2018 to 2021, and COVID‐19 (ICD‐10 *U07.1) and cancer listed from March 2020 to December 2021.^7^ Excess mortality was calculated by comparing the increase in total deaths from the prepandemic (March 2018–December 2019) to pandemic period (March 2020–December 2021). Yearly age‐adjusted mortality rates (AAMR) per 1,000,000 population and average monthly deaths during the prevaccination (March 2020–January 2021) and vaccination (February 2021–December 2021) periods were compared across race/ethnicity groups, sex, and census regions using 95% confidence intervals (CI). Crude mortality rates were compared across various age groups and regions based on urbanization status. Descriptive statistics were used to evaluate the results. Race/ethnicity groups and the different age groups are shown in Table 1. Urbanization status and nine census divisions were included based on the 2013 NCHS Urban–Rural Classification Scheme for Counties and the U.S. Census Bureau, respectively.^8^, ^9^ As CDC WONDER Data is de‐identified and publicly available, this study was deemed exempt from IRB review.

**TABLE 1:** Total deaths, mortality rates, and percentage change across variable sociodemographic groups in 2020 and 2021.

Characteristic	2020 Deaths	2020 AAMR (95% CI)	2021 Deaths	2021 AAMR (95% CI)	Percentage change
Overall	17,392	41.7 (41–42.3)	23,310	56.7 (56–57.5)	36.0%
Sex					
Female	7332	31.3 (30.6–32)	9929	44 (43.1–44.9)	40.6%
Male	10,060	55.9 (54.8–57)	13,381	73.9 (72.6–75.2)	32.2%
Race/ethnicity					
NH American Indian or Alaska Native	151	62.7 (52.4–72.9)	169	66.3 (56–76.6)	5.7%
NH Asian	464	22.9 (20.8–25)	529	25.5 (23.3–27.7)	11.4%
NH Black or African American	2739	67.9 (65.3–70.6)	2908	70.6 (67.9–73.2)	4.0%
Hispanic or Latino	2075	51.1 (48.8–53.3)	2178	51 (48.7–53.2)	−0.2%
NH Native Hawaiian or Other Pacific Islander	28	49.8 (32.5–73)	24	40.3 (25.5–60.4)	−19.1%
NH White	11,823	37.7 (37–38.3)	17,362	57.5 (56.6–58.3)	46.6%
Census region					
Division 1: New England	888	42.8 (39.9–45.6)	817	40.2 (37.4–43)	−6.1%
Division 2: Middle Atlantic	3177	56.5 (54.5–58.5)	3228	57.4 (55.4–59.4)	1.6%
Division 3: East North Central	2981	48.7 (46.9–50.4)	3872	64.6 (62.5–66.7)	32.6%
Division 4: West North Central	1528	54.4 (51.6–57.1)	1509	55.7 (52.9–58.6)	2.4%
Division 5: South Atlantic	3005	33.5 (32.3–34.7)	4618	52.5 (51–54.1)	56.7%
Division 6: East South Central	1116	45.7 (42.9–48.4)	1853	75.7 (72.2–79.2)	65.6%
Division 7: West South Central	1942	44.1 (42.1–46.1)	2941	67.6 (65.1–70.1)	53.3%
Division 8: Mountain	1054	35.2 (33.1–37.4)	1740	58.3 (55.5–61.1)	65.6%
Division 9: Pacific	1701	26.8 (25.5–28.1)	2732	44.2 (42.5–45.9)	64.9%

Urbanization status		Crude mortality rate (95% CI)		Crude mortality rate (95% CI)	
Large central/fringe metro	9439	51.2 (50.2–52.3)	11,098	59.8 (58.7–60.9)	16.8%
Medium/small metro	4910	49.5 (48.1–50.9)	7462	74.5 (72.8–76.2)	50.5%
Nonmetro (micropolitan/noncore)	3043	66.1 (63.8–68.5)	4750	103.1 (100.2–106)	56.0%
Age					
15–24 years	34	0.1 (0.1–0.1)	43	0.1 (0.1–0.1)	0.0%
25–34 years	63	0.1 (0.1–0.2)	120	0.3 (0.2–0.3)	200.0%
35–44 years	206	0.5 (0.4–0.6)	311	0.7 (0.6–0.8)	40.0%
45–54 years	599	1.5 (1.4–1.6)	1070	2.6 (2.5–2.8)	73.3%
55–64 years	2195	5.2 (5–5.4)	3512	8.2 (7.9–8.5)	57.7%
65–74 years	4566	14 (13.6–14.4)	6715	19.9 (19.5–20.4)	42.1%
75–84 years	5330	32.4 (31.5–33.3)	7005	43.2 (42.2–44.2)	33.3%
85+ years	4391	65.9 (64–67.9)	4513	75.5 (73.3–77.7)	14.6%

## 3 RESULTS

### 3.1 Overall changes during the pandemic

**FIGURE 1:**
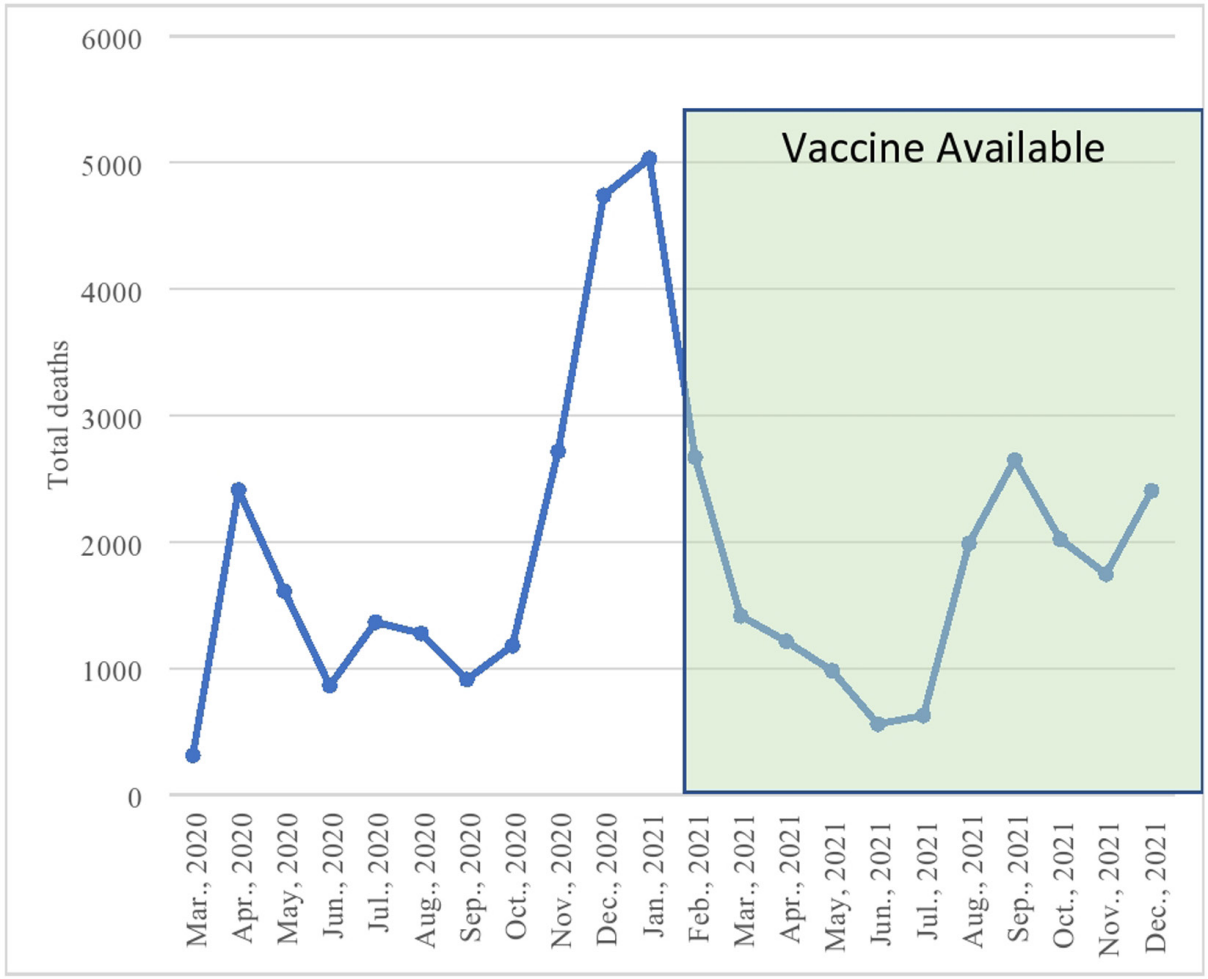
Monthly death totals for patients with COVID‐19 and cancer listed on MCOD from March 2020 to December 2021.

Total deaths in patients with cancer during the pandemic was 1,266,958 which was increased by 4.1%, or 50,264 excess deaths, compared to pre‐pandemic totals. Deaths in patients with both COVID‐19 and cancer listed on MCOD during the pandemic totaled 40,702, or 81.0% of the excess death total in patients with cancer during the pandemic. Yearly totals were 17,392 and 23,310 in 2020 and 2021 respectively. Monthly average deaths decreased by 18.5% in the period of vaccination availability compared to the pre‐vaccination period. (Figure 1).

### 3.2 AAMR comparisons

AAMR in patients with COVID‐19 and cancer on MCOD was 41.7 (95% CI: 41.0–42.3) in 2020 and 56.7 (95% CI: 56.0–57.5) in 2021, an increase of 36%. AAMR was significantly higher in NH American Indian or Alaskan Native, NH Black or African American, and Hispanic or Latino race/ethnicity groups compared to NH White and NH Asian race/ethnicity groups in 2020. In 2021, NH Asian race/ethnicity group had significantly lower AAMR than all other race/ethnicity groups through the course of the pandemic. AAMR was significantly higher in males across both years. (Table 1).

Annual percentage change (APC) in AAMR from 2020 to 2021 for male and female sex increased by 32.2% and 40.6%, respectively. Regarding race/ethnicity groups, NH White had the only significant increase in APC in AAMR of 46.6%. East South Central, Mountain, and Pacific regions had the highest significant increases in APC. (Table 1).

### 3.3 Crude mortality rate comparisons

Regarding urbanization status, rural counties had significantly higher crude mortality rates across both years and the largest increase in APC. Death rates increased with age, and ages 85 years or greater was associated with the highest crude mortality rates across both years. Ages 25–34 years had the largest increase in APC. (Table 1).

## 4 DISCUSSION

In this study, we found mortality in patients with cancer increased by approximately 4%, over 50,000 excess deaths, during the COVID‐19 pandemic. Patients with COVID‐19 and cancer as contributing causes of death accounted for approximately 80%, or 40,000 of these deaths. AAMR in patients with cancer and COVID‐19 were found to be significantly higher in NH Black or African American, Hispanic or Latino, and NH American Indian or Alaskan Native groups in 2020, exacerbating persistent disparities in health outcomes in these groups in the US. This goes in line with known findings which showed that cancer‐associated COVID‐19 mortality was higher in Black and Hispanic groups compared to NH White and NH Asian groups.^3^

NH White had the only significant increases in AAMR in 2021, despite the widespread availability of vaccines, which merits further investigation. AAMR was also significantly higher in males compared to females. This finding correlates with prior research which has shown the risk of mortality in COVID‐19 patients is higher in males compared to females. Cancer patients are also more likely to be male, a significant predictive factor for COVID‐19 mortality.^10^ Males have a slightly higher chance of being diagnosed with an invasive cancer in their lifetime compared to females which may be due to various behavioral, biological, and environmental factors.^11^ Crude mortality rates were highest in rural areas, and this disparity only furthered during 2021. AAMR in those 85 and older was highest, but the greatest increase in mortality from 2020 to 2021 was experienced by those ages 45–64.

Limitations included that we did not utilize underlying cause of death to provide broader comparison across groups. Since the study only utilized MCOD data, it may be difficult to ascertain whether the underlying cause of death is from COVID‐19, cancer, or another underlying condition that worsened the state of either of the two medical conditions. The study also primarily focused on patients with cancer in general and did not investigate trends in specific cancers, stages, subtypes, and treatments. This is important to acknowledge as certain cancers and stages of cancer, such as lung cancer, may have a more aggressive course and be associated with higher rates of mortality whereas other types may have a more indolent course and may be incidentally detected through screening, such as certain grades of prostate cancer.^11^ Such research has previously been explored in brief but merits further detailed investigation as a direction for future research once national data becomes available. Furthermore, AAMR in 2020 may be lower because COVID‐19 did not become widespread until March 2020, which must be considered when using this comparison; however, this method allows for identification of significant differences across groups.

This study found that rates were higher in minority groups, especially in 2020, but NH White patients had a significant increase in 2021 despite widespread vaccination availability. Identification of patients with cancer at higher risk of death from COVID‐19 is important to address disparities in access or care (e.g., vaccination uptake) due to structural racism and other socioeconomic causes to prevent adverse outcomes including mortality. Cancer patients are at an increased risk of developing post‐COVID‐19 complications that may affect various organs; those especially at risk include male gender and patients 65 years and older.^12^

## AUTHOR CONTRIBUTIONS

**Bhaghyasree Jambunathan:** Conceptualization (lead); investigation (equal); methodology (equal); project administration (equal); writing – original draft (lead); writing – review and editing (equal). **Jacob Lang:** Conceptualization (equal); formal analysis (lead); investigation (equal); methodology (equal); visualization (lead); writing – original draft (equal); writing – review and editing (equal). **Malik Mays:** Conceptualization (equal); investigation (equal); writing – original draft (equal); writing – review and editing (equal). **Obi Ekwenna:** Conceptualization (equal); investigation (equal); methodology (equal); project administration (equal); supervision (lead); writing – original draft (equal); writing – review and editing (equal).

## CONFLICT OF INTEREST STATEMENT

The authors have no conflicts of interest to disclose.

## ETHICS STATEMENT

No patient consent or ethics approval required because the data analyzed is publicly available.

## Data Availability

The data underlying this article is available in the CDC Wide‐Ranging Online Data for Epidemiologic Research Database at https://wonder.cdc.gov/mcd‐icd10‐expanded.html.
